# Endotoxin Producers Overgrowing in Human Gut Microbiota as the Causative Agents for Nonalcoholic Fatty Liver Disease

**DOI:** 10.1128/mBio.03263-19

**Published:** 2020-02-04

**Authors:** Na Fei, Aurélia Bruneau, Xiaojun Zhang, Ruirui Wang, Jinxing Wang, Sylvie Rabot, Philippe Gérard, Liping Zhao

**Affiliations:** a Université Paris-Saclay, INRAE, AgroParisTech, Micalis Institute, Jouy-en-Josas, France; b State Key Laboratory of Microbial Metabolism, School of Life Sciences and Biotechnology, Shanghai Jiao Tong University, Shanghai, People’s Republic of China; c Ministry of Education Key Laboratory for Systems Biomedicine, Shanghai Centre for Systems Biomedicine, Shanghai Jiao Tong University, Shanghai, People’s Republic of China; University of Hawaii at Manoa

**Keywords:** gut inflammation, intestinal microbiology, fatty liver, nonalcoholic steatohepatitis, gut inflammation

## Abstract

Gut microbiota-derived endotoxin has been linked to human nonalcoholic fatty liver disease (NAFLD), but the specific causative agents and their molecular mechanisms remain elusive. In this study, we investigated whether bacterial strains of endotoxin-producing pathogenic species overgrowing in obese human gut can work as causative agents for NAFLD. We further assessed the role of lipopolysaccharide (LPS)-Toll-like receptor 4 (TLR4) cross talk in this pathogenicity. Nonvirulent strains of Gram-negative pathobionts were isolated from obese human gut and monoassociated with C57BL/6J germfree (GF) mice fed a high-fat diet (HFD). Deletion of *waaG* in the bacterial endotoxin synthetic pathway and knockout of TLR4 in GF mice were used to further study the underlying mechanism for a causal relationship between these strains and the development of NAFLD. Three endotoxin-producing strains, Enterobacter cloacae B29, Escherichia coli PY102, and Klebsiella pneumoniae A7, overgrowing in the gut of morbidly obese volunteers with severe fatty liver, induced NAFLD when monoassociated with GF mice on HFD, while HFD alone did not induce the disease in GF mice. The commensal Bacteroides thetaiotaomicron (ATCC 29148), whose endotoxin activity was markedly lower than that of *Enterobacteriaceae* strains, did not induce NAFLD in GF mice. B29 lost its proinflammatory properties and NAFLD-inducing capacity upon deletion of the *waaG* gene. Moreover, E. cloacae B29 did not induce NAFLD in TLR4-deficient GF mice. These nonvirulent endotoxin-producing strains in pathobiont species overgrowing in human gut may work as causative agents, with LPS-TLR4 cross talk as the most upstream and essential molecular event for NAFLD.

## Abstract (precis)

Recent studies have reported a link between gut microbiota and nonalcoholic fatty liver disease (NAFLD), showing that germfree (GF) mice do not develop metabolic syndromes, including NAFLD. However, the specific bacterial species causing NAFLD, as well as their molecular cross talk with the host for driving liver disease, remain elusive. Here, we found that nonvirulent endotoxin-producing strains of pathogenic species overgrowing in obese human gut can act as causative agents for induction of NAFLD and related metabolic disorders. The cross talk between endotoxin from these specific producers and the host’s TLR4 receptor is the most upstream and essential molecular event for inducing all phenotypes in NAFLD and related metabolic disorders. These nonvirulent endotoxin-producing strains of gut pathogenic species overgrowing in human gut may collectively become a predictive biomarker or serve as a novel therapeutic target for NAFLD and related metabolic disorders.

## INTRODUCTION

Nonalcoholic fatty liver disease (NAFLD) has become the most common cause of chronic liver disease strongly associated with a high risk of obesity and type 2 diabetes (1). During the past decade, evidence for cross talk between the gut microbiome, the liver, the immune system, and metabolism has emerged, suggesting that the resident microbiota has emerged as an important player in the development of NAFLD (2).

Recently, causal evidence for gut microbiota involvement in the pathogenesis of NAFLD has been shown in mice and in humans by transplanting the whole gut microbiota to germfree (GF) mice (3, 4). High-fat diet (HFD)-fed GF mice exhibit lower levels of lipids in the liver than conventionally housed mice (3, 4), indicating that HFD is not sufficient for disease development. Gut microbiota from mice that developed fasting hyperglycemia and insulinemia, but not from healthy mice, led to the development of NAFLD in recipient GF mice (5). Fecal transplantation from human donors with hepatic steatosis triggered a rapid development of hepatic steatosis in mice (6). Gut microbiota from obese humans, but not from the same donor after weight loss, induced the onset of hepatic steatosis through modulation of lipid metabolism transcriptional profiles in GF mice (7). HFD-fed GF mice inoculated with microbiota of nonalcoholic steatohepatitis (NASH) patients, rather than healthy donors, showed an exacerbated NAFLD phenotype, as manifested by increased liver steatosis and inflammation (8). Collectively, these pieces of evidence indicate a causal role of the gut microbiota in the development of NAFLD (9).

NAFLD has been associated with increased levels of specific Gram-negative bacterial species, including *Proteobacteria*, *Enterobacteria*, *Escherichia* (10, 11), and *Bacteroides* (11, 12). A higher representation of *Gammaproteobacteria* and *Epsilonproteobacteria* was also seen in children with NAFLD than in healthy lean and obese children (13). These studies indicated an association between Gram-negative bacteria and progression of NAFLD. However, strains from the same species may have different functions due to the still high genetic diversity (14). Moreover, these studies failed to causally identify microorganisms that differentiate individuals with the disease and the control population.

Activation of innate immunity is associated with the development of NAFLD (15). Gut-derived toxins, such as endotoxins, are suggested to have causative roles in liver inflammation, as well as the onset and progression of chronic liver diseases (16). Serum LPS levels are increased in patients with hepatic steatosis caused by total parenteral nutrition or intestinal bypass (17, 18). In mice on standard laboratory chow, continuous subcutaneous infusion of low-dose LPS results in hepatic steatosis, hepatic insulin resistance, and hepatic weight gain (19). In addition, an intraperitoneal injection of LPS exacerbates liver injury in mice fed a methionine-choline-deficient diet (20). The LPS-binding protein (LBP)-CD14 complex activates Toll-like receptor 4 (TLR4), triggering an essential inflammatory cascade in the progression of NAFLD (21, 22). These data indicate that the liver is the main target of LPS, and LPS-TLR4 is a key pathway. However, the role of specific bacteria and their bioactive molecules and their contribution to the inflammation-driven process in NAFLD remain to be definitively identified and demonstrated as the cause.

We previously showed that one nonvirulent, endotoxin-producing strain, B29, from the sepsis-inducing species Enterobacter cloacae, overgrowing in the gut of an obese human, can induce obesity when monocolonized in GF mice on HFD (23). The primary aim of the present study was to establish a definitive cause-effect link between the endotoxin producers enriched in obese subjects and NAFLD development. We also want to further elucidate the molecular cross talk between these bacteria and their host in the initiation and progression of this disease.

## RESULTS

### Human case study. (i) Volunteer 1.

Volunteer 1 was a 26-year-old morbidly obese man (weight, 174.8 kg; height, 172.5 cm; body mass index [BMI], 58.78 kg/m^2^; Taiyuan city, Shanxi Province, China) with coexisting metabolic syndrome (23). Besides obesity, this volunteer also had severe fatty liver, as observed by ultrasonography, with concomitant markedly increased levels of serum concentrations of aspartate aminotransferase (122 U liter^−1^), alanine aminotransferase (97 U liter^−1^), and gamma-glutamyl transferase (168 U liter^−1^) (23).

This volunteer was given a diet composed of whole grains, traditional Chinese medicine, and prebiotics (WTP diet) for 23 weeks. He lost 30.1 kg after 9 weeks and 51.4 kg after 23 weeks on the diet, with continued amelioration of hyperinsulinemia, hyperglycemia, hypertension, and liver function until most metabolic parameters improved to normal ranges. *Enterobacter* made up 35% of the gut bacteria in volunteer 1 at the beginning, and after 9 weeks on the WTP diet, the *Enterobacter* population in the volunteer’s gut reduced to 1.8% and became undetectable by the end of the 23-week trial (23). Details were presented in our previous study. Enterobacter cloacae B29 was isolated from the baseline fecal sample of this volunteer as the most abundant pathobiont. Klebsiella pneumoniae A7 was isolated from the same volunteer’s gut as the second most abundant pathobiont (23).

### (ii) Volunteer 2.

Volunteer 2 was a 3-year-old morbidly obese girl (Chinese; initial body weight, 46 kg; Shanxi Province, China) with coexisting metabolic syndrome. Escherichia coli was detected as 40% of her gut microbiota, as shown using sequencing of the V3 region of the 16S rRNA gene (data not published). She lost 16 kg after 20 weeks with alleviated fatty liver phenotype on the WTP diet, and the E. coli population was reduced to nondetectable soon after she was on the diet. Escherichia coli PY102 was isolated from the feces of volunteer 2 via a sequence-guided isolation scheme (23 and data not published).

### E. cloacae B29 acts as a causative agent for NAFLD induction in GF mice under HFD feeding.

**FIG 1:**
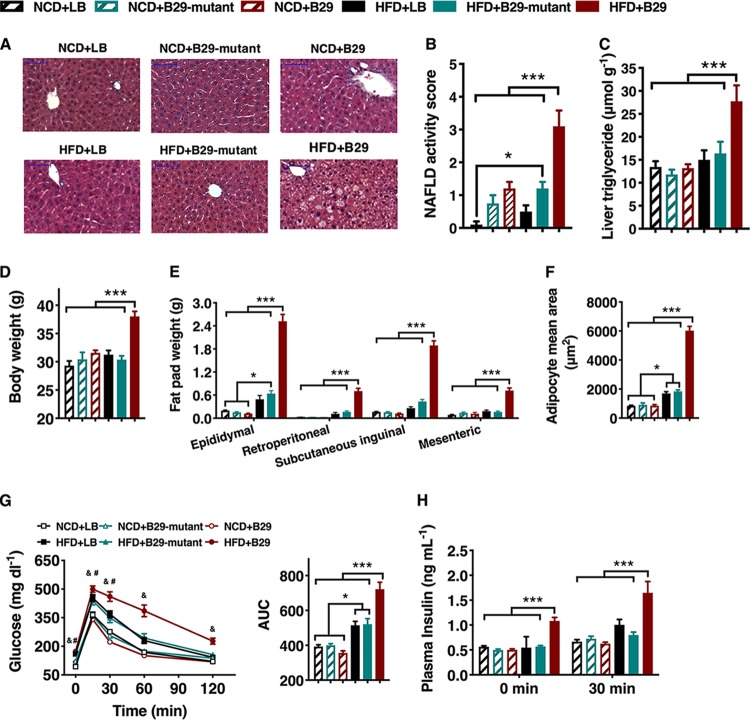
E. cloacae B29-dependent induction of NAFLD in GF mice under HFD feeding requires endotoxin expression. Germfree (GF) mice were fed either normal chow diet (NCD) or high-fat diet (HFD) and inoculated with either Luria-Bertani (LB), wild-type E. cloacae B29, or the endotoxin-lacking *waaG* mutant strain (data were collected at the end of 15 weeks after inoculation). (A) Liver histology (hematoxylin and eosin stain). Scale bar, 50 μm. (B) NAFLD activity score. (C) Liver triglyceride. (D) Body weight. (E) Mass of epididymal, mesenteric, subcutaneous inguinal, and retroperitoneal fat pads. (F) Adipocyte mean area. (G) Oral glucose tolerance test (OGTT) and the area under the curve (AUC) for the plasma glucose concentration (*P < *0.05; &, HFD+B29 versus other groups; #, HFD+LB and HFD+B29-mutant versus NCD groups). (H) Plasma insulin concentration before (fasting) and 30 min after oral glucose load. Data shown are means ± SEM (*n* = 5 to 10). *, *P < *0.05; **, *P < *0.01; ***, *P < *0.001.

**FIG 2:**
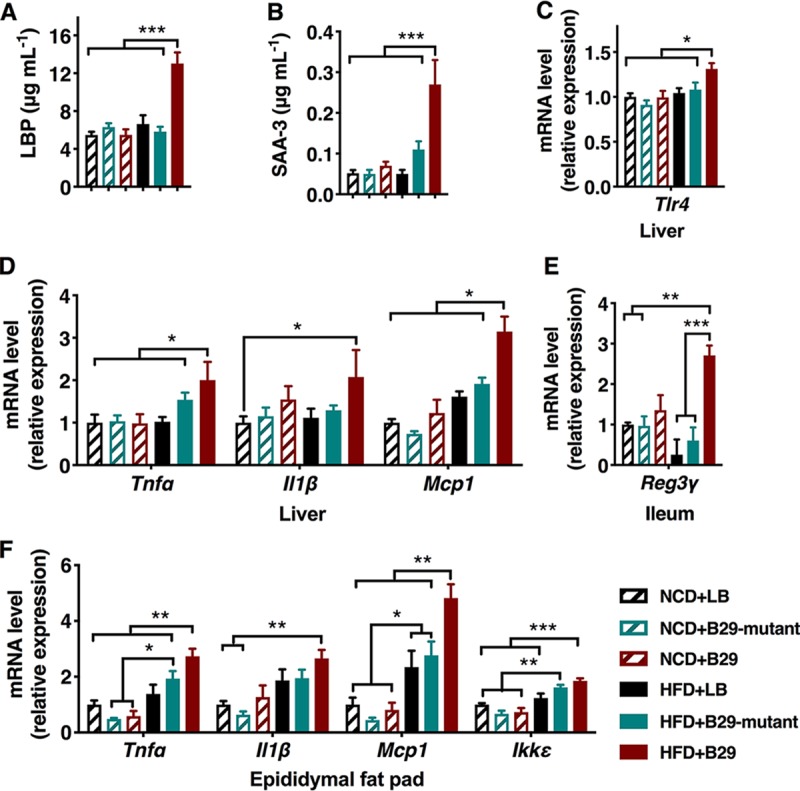
E. cloacae B29 induction of systemic and local inflammation in HFD-fed NAFLD GF mice is dependent upon endotoxin expression. GF mice were fed either normal chow diet (NCD) or high-fat diet (HFD) and inoculated with either Luria-Bertani broth (LB), wild-type E. cloacae B29, or the endotoxin-lacking *waaG* mutant strain (data were collected at the end of 15 weeks after inoculation). (A) ELISA of serum LPS-binding protein (LBP). (B) ELISA of serum amyloid A (SAA-3). (C) RT-qPCR analysis of expression of the *Tlr4* gene in the liver. (D to F) RT-qPCR analysis of expression of the *Tnfα*, *Il1β*, *Mcp1*, *Ikkε*, and *Reg3γ* genes in the liver (D), ileum (E), and epididymal fat pad (F). All mRNA quantification data were normalized against the housekeeping gene. Gene expression levels were expressed as values relative to those of the control group (NCD+LB). *Tlr4*, toll-like receptor 4 gene; *Tnfα*, tumor necrosis factor-α gene; *Il1β*, interleukin-1β gene; *Mcp1*, monocyte chemoattractant protein-1 gene; *Ikkε*, I kappa B kinase epsilon gene; *Reg3γ*, c-type lectin regenerating islet-derived protein 3-γ. Data shown are means ± SEM (*n* = 5 to 10). *, *P < *0.05; **, *P < *0.01; ***, *P < *0.001.

To test whether nonvirulent endotoxin-producing strains of gut pathogenic species overgrowing in obese human gut can act as causative agents for NAFLD induction, we inoculated 10^9^ to 10^10^ cells of E. cloacae B29 into C57BL/6J GF mice fed either a normal chow diet (NCD) or HFD. E. cloacae B29 achieved a population level of between 10^9^ and 10^10^ cells/g of feces (see Fig. S1 in the supplemental material). At the end of the trial, NCD-fed mice colonized with wild-type E. cloacae B29 showed no significant difference in the features of NAFLD (Fig. 1A to C) and gained a similar amount of body weight (Fig. 1D to F and Fig. S2) compared to the NCD-fed control mice. On the contrary, HFD-fed mice colonized by E. cloacae B29 (termed HFD+B29), but not the HFD-fed GF control mice, were characterized by significantly enhanced hepatic steatosis and NAFLD activity scores (Fig. 1A to C), among other obesity-related phenotypes. These mice also displayed significantly higher body weight than the HFD-fed GF control mice (Fig. 1D and Fig. S2) despite possessing the smallest cecum of all the mice (GF mice normally possess a significantly bigger cecum than the conventional mice) (Fig. S2) (24). The HFD+B29 mice also exhibited a significant increase in fat mass (epididymal, mesenteric, subcutaneous inguinal, and retroperitoneal fat pads) (Fig. 1E and F) and blood lipid concentration (Fig. S2) compared to levels of all other groups. Moreover, the increases in serum leptin concentration and epididymal fat pad *Leptin* gene expression level were observed in HFD+B29 mice (Fig. S2). The obese HFD+B29 mice also exhibited statistically significant elevations in blood glucose and insulin levels (both fasted and 30 min postchallenge) and a marked decrease in glucose tolerance relative to all other groups (Fig. 1G and H), consistent with features of insulin resistance. We also found significantly increased expression of fatty acid synthase (*Fas*) and peroxisome proliferator-activated receptor-gamma (*Pparγ*) in the liver and fat pad and decreased expression of fasting-induced adipose factor (*Angptl4*) in the liver and ileum of HFD+B29 mice (Fig. S2), indicating a disrupted lipometabolism favoring synthesis or storage but not removal of fat. Inflammation plays a critical role in NAFLD development (25). Elevated serum levels of LBP and proinflammatory cytokine serum amyloid A protein (SAA-3) and increased expression of the *Tlr4* gene in the liver (Fig. 2A and B) were detected in the HFD+B29 mice but not the HFD-fed GF control mice (Fig. 2A and B). In agreement with these results, gene expression analysis revealed significantly elevated levels of mRNA corresponding to the proinflammatory cytokines tumor necrosis factor alpha (*Tnfα*), interleukin-1β (*Il1β*), interleukin-6 (*Il6*), monocyte chemoattractant protein-1 (*Mcp1*-*Ccl2*), and inhibitor of κB kinase epsilon (*Ikkε*) in the liver and adipose tissue and c-type lectin-regenerating islet-derived protein 3-γ (*Reg3γ*) in the gut of HFD+B29 mice compared to those of the other groups (Fig. 2D to F). In summary, all features of NAFLD and metabolic abnormalities were observed only in HFD+B29 mice. This indicates that E. cloacae B29 works as a causative agent for NAFLD while HFD works as the necessary environmental factor, which itself does not induce the disease.

10.1128/mBio.03263-19.1 FIG S1 Population levels of Enterobacter cloacae B29 or the B29Δ*waaG* mutant in the feces of monoassociated gnotobiotic mice. Data are shown as means ± SEM (*n* = 5 to ∼10). *, *P* < 0.05; **, *P* < 0.01. NCD, normal chow diet; HFD, high-fat diet. Download FIG S1, TIF file, 0.4 MB. Copyright © 2020 Fei et al. 2020 Fei et al. This content is distributed under the terms of the Creative Commons Attribution 4.0 International license.

10.1128/mBio.03263-19.2 FIG S2 E. cloacae B29 *waaG* mutant loses the capacity to induce obesity in GF mice under HFD feeding. (A) Growth curves. (B) Cecum content weight to body weight. (C to F) Serum lipid concentration. (G) Serum leptin concentration adjusted for body weight. (H to K) RT-qPCR analysis of the gene expression of *Fas*, *Pparγ*, *Scd1*, and *Angptl4* (*Fiaf*) in the liver (H), *Leptin* expression in the epididymal fat pad (I), *Fas* in the epididymal fat pad (J), or *Angptl4* (*Fiaf*) in the ileum (K). (L and M) Total food intake under NCD (L) or HFD (M) feeding. (N) RT-qPCR analysis of expression of *Glp1*, *Pyy*, and *Ghrelin* in the ileum. All mRNA quantification data were normalized against the housekeeping gene. Gene expression levels were expressed as values relative to those of the control group (NCD+LB). HDL, high-density lipoprotein; *Fas*, fatty acid synthase gene; *Pparγ*, peroxisome proliferator-activated receptor-gamma gene; *Scd1*, stearoyl-coenzyme A desaturase 1 gene; *Angptl4* (*Fiaf*), fasting-induced adipose factor gene. Data are shown as means ± SEM (*n* = 5 to 10). HFD+B29 versus other groups. GF, germfree. *, *P < *0.05; **, *P < *0.01; ***, *P < *0.001. Download FIG S2, TIF file, 1.1 MB. Copyright © 2020 Fei et al. 2020 Fei et al. This content is distributed under the terms of the Creative Commons Attribution 4.0 International license.

### E. cloacae B29 induction of systemic and local inflammation in HFD-fed NAFLD GF mice is dependent upon endotoxin expression.

To demonstrate that the production of proinflammatory LPS endotoxin from the gut pathogenic species is essential for inducing NAFLD and subsequent metabolic complications in mice, we deleted the *waaG* gene of E. cloacae B29 (26). *waaG* is involved in the synthesis of LPS and encodes a protein that joins the outer core and O-antigen parts of the LPS molecule by catalyzing the transfer of a glucose moiety from the donor nucleotide sugar UDP-α-d-glucose to the l-glycero-d-manno-heptose II of the inner core of LPS (26, 27). The LPS endotoxin activity of the E. cloacae B29Δ*waaG* mutant was reduced approximately 600-fold from that of B29 (Fig. S3). Thus, the B29Δ*waaG* mutant essentially lost its proinflammatory capacity.

10.1128/mBio.03263-19.3 FIG S3 Endotoxin activity of Enterobacter cloacae B29 wild-type strain and B29Δ*waaG* mutant based on *Limulus* amebocyte lysate test. E. coli 055:B5 LPS (Sigma) was used as a positive control. Data are shown as means ± SEM (*n* = 3). ***, *P < *0.001. LPS pellet from each strain was dissolved in 10 mM Tris-HCl buffer (pH 8.0). Download FIG S3, TIF file, 0.1 MB. Copyright © 2020 Fei et al. 2020 Fei et al. This content is distributed under the terms of the Creative Commons Attribution 4.0 International license.

We then compared the NAFLD-inducing effect of the B29Δ*waaG* mutant to that of its wild-type parent in C57BL/6J GF mice fed on HFD. The B29Δ*waaG* strain also achieved a population level of between 10^9^ and 10^10^ cells/g of feces in the gut of gnotobiotic mice (Fig. S1). Intriguingly, at the end of the trial, all features of NAFLD and metabolic abnormalities observed in HFD+B29 mice, including degenerated hepatocytes, increased NAFLD activity score, body weight, fat pad mass, and blood lipid, glucose, and insulin levels, were not observed in mice upon colonization with the *waaG* mutant strain (HFD+mutant) (Fig. 1 and Fig. S2).

We next asked whether deletion of *waaG* gene in E. cloacae B29 indeed failed to induce proinflammatory responses in mice. As expected, the elevated serum levels of LBP and proinflammatory cytokine SAA-3 and increased expression of the *Tlr4* gene in the liver of HFD+B29 mice (Fig. 2D) were not detected in the HFD+mutant mice (Fig. 2A and B). In agreement with these results, significantly elevated levels of mRNA corresponding to the proinflammatory cytokines *Tnfα*, *Il1β*, *Il6*, *Mcp1*-*Ccl2*, and *Ikkε* in the liver and adipose tissue and *Reg3γ* in the gut of HFD+B29 mice were completely absent in HFD+mutant littermates (Fig. 2E to G). Taking these findings together, the B29Δ*waaG* mutant does not induce systemic inflammation or the development of NAFLD in HFD-fed GF mice. This indicates that the proinflammatory capacity of its LPS endotoxin is essential to the NAFLD-inducing property of E. cloacae B29.

### Absence of TLR4 in mice prevented NAFLD induced by the LPS-producing gut opportunistic pathobiont Enterobacter cloacae B29.

**FIG 3:**
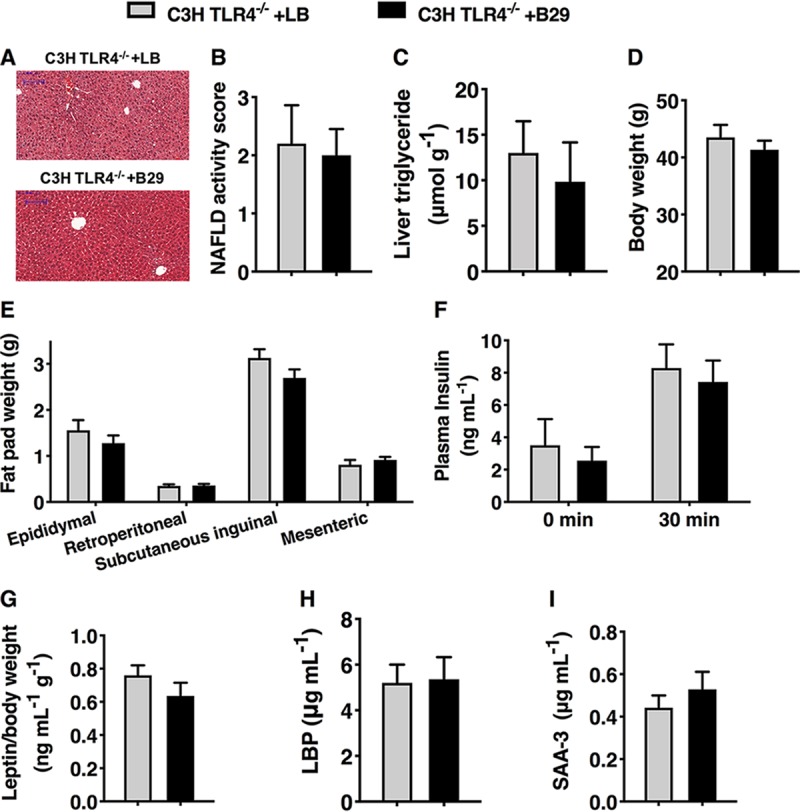
Absence of TLR4 in mice prevented NAFLD induced by the LPS-producing gut opportunistic pathobiont Enterobacter cloacae B29 (data were collected at the end of 15 weeks after inoculation). (A) Liver histology (hematoxylin and eosin stain) of TLR4 mutant (TLR4^−/−^) mice with or without B29 at the end of the trial. Scale bar, 100 μm. (B) NAFLD activity score. (C) Body weight. (D) Mass of epididymal, mesenteric, subcutaneous inguinal, and retroperitoneal fat pads of TLR4^−/−^mice with or without B29. (E) Plasma insulin concentration before (fasting) and 30 min after oral glucose load in TLR4^−/−^ mice with or without B29. (F) ELISA of serum leptin in TLR4^−/−^ mice with or without B29 (after adjustment for body weight). (G) ELISA of serum LBP in TLR4^−/−^ mice with or without B29. (H) ELISA of serum amyloid A (SAA-3) in TLR4^−/−^ mice with or without B29. Data shown are means ± SEM (*n* = 5 to 9). *, *P < *0.05; **, *P < *0.01; ***, *P < *0.001.

We investigated whether the endotoxin-producing pathobiont E. cloacae B29 induces NAFLD in C3H/HeN GF mice with or without TLR4 deficiency to decipher if B29 acts through the LPS-TLR4 signaling pathway and whether its capacity to induce NAFLD is a common phenomenon independent of the genetics of the mouse lines. We analyzed the NAFLD-inducing property of E. cloacae B29 with HFD-fed C3H/HeN GF mice with or without TLR4 deficiency. E. cloacae B29 achieved a population level of between 10^9^ and 10^10^ cells/g of feces, with no significant differences between the gnotobiotic mice with different genetic backgrounds (Fig. S4). HFD-fed wild-type C3H/HeN mice colonized with B29 showed markedly degenerated hepatocytes, a significant increase in NAFLD activity score, obesity, and an insulin-resistant phenotype, unlike the corresponding HFD-fed GF control mice, after 15 weeks of HFD treatment (Fig. S5 and S6). Wild-type C3H/HeN mice colonized with B29 showed significant increases in serum endotoxin load or local and systemic inflammatory levels compared to those of the corresponding GF controls (Fig. S4 and S5). GF TLR4^−/−^ C3H/HeN mice colonized by B29, however, did not develop any NAFLD or other metabolic disease features not seen in noncolonized controls (Fig. 3A to G and Fig. S7). Importantly, E. cloacae B29 also failed to induce an endotoxin response or increased downstream inflammatory cytokine production in the GF TLR4^−/−^ mice compared to that of the corresponding GF control (Fig. 3H and I and Fig. S4). These results indicate that E. cloacae B29 is also able to induce NAFLD in C3H/HeN mice and that its NAFLD-inducing capacity requires an intact TLR4 receptor from the host side. TLR4 has been known to be involved in the development of NAFLD (28) and metabolic syndrome (29–32). This study demonstrates that the cross talk between endotoxin from specific producers overgrowing in the obese human gut and the host’s TLR4 receptor is the most upstream and essential process for inducing NAFLD. The NAFLD phenotype induced by E. cloacae B29 is a common phenomenon independent of the genetics of the mouse lines.

10.1128/mBio.03263-19.4 FIG S4 Local inflammation in the Enterobacter cloacae strain B29 monoassociated C3H/HeN TLR4 WT and GF control mice (A) and the B29 mono-associated C3H/HeN TLR4 null mutant and GF control mice (B) under HFD feeding (data were collected at the end of 15 weeks after inoculation). (A, a) Population levels of Enterobacter cloacae B29 in gnotobiotic mice. (b and c) RT-qPCR analysis of expression of *Tlr4* and *Cd14* in the liver (b) and epididymal fat pad (c). (d to f) RT-qPCR analysis of expression of *Tnfα*, *Mcp1*, *Il1β*, and *Ikkε* in the liver (d), epididymal fat pad (e), or ileum (f). (B, a) Population levels of Enterobacter cloacae B29 in gnotobiotic mice. (b and c) RT-qPCR analysis of expression of *Cd14* and *Lbp* in the liver (b) and ileum (c). (d to f) RT-qPCR analysis of expression of *Tnfα*, *Il1β*, *Mcp1*, *Il6*, *Ikkε*, and *Reg3γ* in the liver (d), epididymal fat pad (e), or ileum (f). All mRNA quantification data were normalized against the housekeeping gene. Gene expression levels were expressed as values relative to those of the control group (C3H TLR4^−/−^ +LB). *Lbp*, LPS-binding protein gene; *Tlr4*, toll-like receptor 4 gene; *Tnfα*, tumor necrosis factor-α gene; *Il1β*, interleukin-1β gene; *Il6*, interleukin-6 gene; *Mcp1*, monocyte chemoattractant protein-1 gene; *Ikkε*, I kappa B kinase epsilon gene; *Reg3γ*, c-type lectin regenerating islet-derived protein 3-γ gene. TLR4^−/−^, Toll-like receptor 4 mutant mice; TLR4 WT, Toll-like receptor 4 wild-type mice. Data are shown as means ± SEM (*n* = 5 to 6). Download FIG S4, TIF file, 1.2 MB. Copyright © 2020 Fei et al. 2020 Fei et al. This content is distributed under the terms of the Creative Commons Attribution 4.0 International license.

10.1128/mBio.03263-19.5 FIG S5 HFD-fed wild-type C3H/HeN mice developed NAFLD after being colonized by the LPS-producing gut opportunistic pathobiont Enterobacter cloacae B29 (data were collected at the end of 15 weeks after inoculation). (A) Liver histology (hematoxylin and eosin stain) of Toll-like receptor 4 (TLR4) wild-type (WT) mice with or without B29 at the end of the trial. Scale bar, 100 μm. (B) NAFLD activity score. (C) Body weight. (D) Mass of epididymal, mesenteric, subcutaneous inguinal, and retroperitoneal fat pads of TLR4 WT mice with or without B29. (E) Plasma insulin concentration before (fasting) and 30 min after oral glucose load in TLR4 WT mice with or without B29. (F) Enzyme-linked immunosorbent assay (ELISA) of serum leptin in TLR4 WT mice with or without B29 (after adjustment for body weight). (G) ELISA of serum LBP in TLR4 WT mice with or without B29. (H) ELISA of serum amyloid A (SAA-3) in TLR4 WT mice with or without B29. Data shown are means ± SEM (*n* = 5 to 9). *, *P < *0.05; **, *P < *0.01; ***, *P < *0.001. Download FIG S5, TIF file, 1.3 MB. Copyright © 2020 Fei et al. 2020 Fei et al. This content is distributed under the terms of the Creative Commons Attribution 4.0 International license.

10.1128/mBio.03263-19.6 FIG S6 Enterobacter cloacae strain B29 induced obesity after associated with GF C3H/HeN TLR4 WT mice under HFD feeding. (A) Growth curves. (B and C) Adipocyte mean area (B) and number (C) (epididymal fat pad, hematoxylin- and eosin-stained sections, ×200). (D) Cecum content weight. (E) Cecum content weight (percentage of the body weight). (F) Oral glucose tolerance test (OGTT) and the areas under the curve (AUC). (G) Serum leptin concentration adjusted for body weight. (H to J) RT-qPCR analysis of the gene expression of AccI, *Fas*, *Srebp1*, *Pparγ*, *Scd1*, *Angptl4* (*Fiaf*), *Fabp1*, *Gpr43*, and *Gpr41* in the liver (H), epididymal fat pad (I), or ileum (J). All mRNA quantification data were normalized against the housekeeping gene. Gene expression levels were expressed as values relative to those of the control group (C3H TLR4 WT+LB). AccI, acetyl-CoA carboxylase-1 gene; *Fas*, fatty acid synthase gene; *Pparγ*, peroxisome proliferator-activated receptor-gamma gene; *Srebp1*, sterol regulatory element-binding protein 1 gene; *Scd1*, stearoyl-CoA desaturase 1 gene; *Angptl4* (*Fiaf*), fasting-induced adipose factor gene; *Fabp*, fatty acid-binding proteins gene; *Gpr43*, G protein-coupled receptor 43 gene; *Gpr41*, G protein-coupled receptor 41 gene. Data are shown as means ± SEM (*n* = 8 to 9). *, *P < *0.05; **, *P < *0.01; ***, *P < *0.001. Download FIG S6, TIF file, 1.0 MB. Copyright © 2020 Fei et al. 2020 Fei et al. This content is distributed under the terms of the Creative Commons Attribution 4.0 International license.

10.1128/mBio.03263-19.7 FIG S7 Enterobacter cloacae strain B29 lost the capacity to induce obesity after being monoassociated with GF C3H/HeN TLR4 null mutant under HFD feeding. (A) Growth curves. (B and C) Adipocyte mean area (B) and number (C) (epididymal fat pad, hematoxylin- and eosin-stained sections, ×200). (D) Cecum content weight. (E) Cecum content weight (percentage of the body weight). (F) Oral glucose tolerance test (OGTT) and the areas under the curve (AUC). (G) Serum leptin concentration adjusted for body weight. (H to J) RT-qPCR analysis of the gene expression of AccI, *Fas*, *Srebp1*, *Pparγ*, *Lpl*, *Scd1*, *Angptl4* (*Fiaf*), *Fabp*, *Cd36*, *Vdr*, *Glut*, *Gpr43*, and *Gpr41* in the liver (H), epididymal fat pad (I), or ileum (J). All mRNA quantification data were normalized against the housekeeping gene. Gene expression levels were expressed as values relative to those of the control group (C3H TLR4^−/−^ +LB). AccI, acetyl-CoA carboxylase-1 gene; *Fas*, fatty acid synthase gene; *Pparγ*, peroxisome proliferator-activated receptor-gamma gene; *Srebp1*, sterol regulatory element-binding protein 1 gene; *Scd1*, stearoyl-CoA desaturase 1 gene; *Angptl4* (*Fiaf*), fasting-induced adipose factor gene; *Lpl*, lipoprotein lipase gene; *Fabp*, fatty acid-binding proteins gene; *Vdr*, vitamin D receptor gene; *Glut*, glucose transporter gene; *Gpr43*, G protein-coupled receptor 43 gene; *Gpr41*, G protein-coupled receptor 41 gene. Data are shown as means ± SEM (*n* = 5 to 6). Download FIG S7, TIF file, 1.1 MB. Copyright © 2020 Fei et al. 2020 Fei et al. This content is distributed under the terms of the Creative Commons Attribution 4.0 International license.

### Gram-negative bacteria producing LPS with different endotoxin activity levels showed different capacities to induce NAFLD in HFD-fed GF mice.

**FIG 4:**
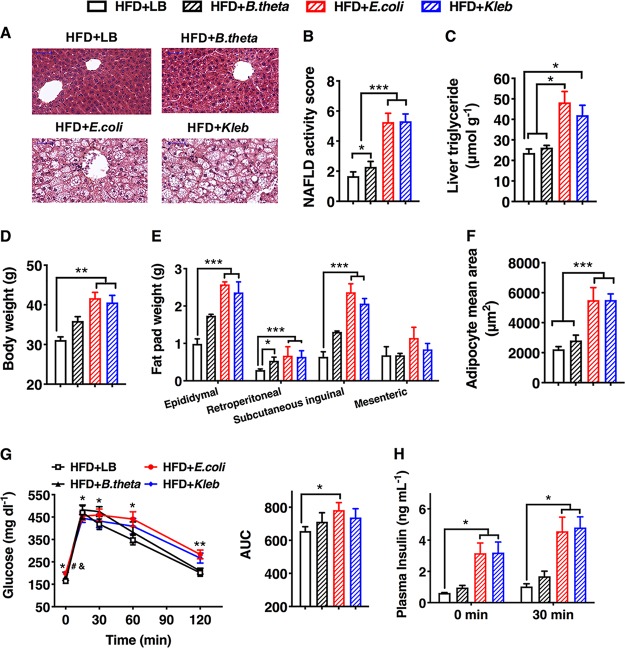
Gram-negative bacteria producing LPS with different endotoxin activity levels showed different capacities to induce NAFLD in HFD-fed GF mice (data were collected at the end of 15 weeks after inoculation). (A) Liver histology (hematoxylin and eosin stain). Scale bar, 50 μm. (B) NAFLD activity score. (C) Liver triglyceride. (D) Body weight. (E) Mass of epididymal, mesenteric, subcutaneous inguinal, and retroperitoneal fat pads. (F) Adipocyte mean area. (G) Oral glucose tolerance test (OGTT) and the area under the curve (AUC) for the plasma glucose concentration (*P* < 0.05; #, HFD+LB versus HFD+B.*theta*; &, HFD+LB versus HFD+*Kleb*; *, HFD+LB versus HFD+*E.coli*). (H) Plasma insulin concentration before (fasting) and 30 min after oral glucose load. Data shown are means ± SEM (*n* = 7 to 16). *, *P < *0.05; **, *P < *0.01; ***, *P < *0.001.

We next hypothesized that this ability to induce inflammation and NAFLD is a more general property of nonvirulent proinflammatory, LPS-producing strains of gut pathogenic species. To test this, we examined the NAFLD-inducing capacity of two other Gram-negative bacteria isolated from the gut of obese volunteers (see Materials and Methods for a detailed description): Klebsiella pneumoniae A7 (23) and Escherichia coli PY102. K. pneumoniae A7 was isolated from the same volunteer’s gut as the second most abundant pathobiont (23). Escherichia coli was detected as 40% of another obese volunteer’s gut microbiota, as shown using sequencing of the V3 region of the 16S rRNA gene (data not published). She lost 16 kg after 20 weeks with alleviated fatty liver phenotype on the WTP diet, and the E. coli population was reduced to nondetectable levels soon after she was on the diet. E. coli PY102 was isolated from the feces of this volunteer via a sequence-guided isolation scheme (23 and data not published). K. pneumoniae A7 and E. coli PY102 showed strong endotoxin activity, as determined by the *Limulus* amebocyte lysate (LAL) test (Fig. S8) (33). HFD-fed GF C57BL/6J mice inoculated with K. pneumoniae A7 (HFD+*Kleb*) or E. coli PY102 (HFD*+E.coli*), reaching a similar bacterial population in the feces (Fig. S8), showed markedly degenerated hepatocytes, significant increase in NAFLD activity score, and associated metabolic syndrome features after 15 weeks of HFD treatment, in contrast to HFD-fed uninfected control mice (Fig. 4A to H and Fig. S9). In addition, HFD+*Kleb* and HFD*+E.coli* mice also displayed a low-grade proinflammatory state, as revealed by markedly increased serum levels of LBP and SAA-3 and high expression levels of certain inflammatory markers, such as *Tnfα* and *Mcp-1*, in the liver, fat pad, and ileum, in contrast to the GF controls (Fig. S8). Taken together, these data suggest that nonvirulent strains of the gut pathogenic species that produce LPS with high endotoxin activity can induce NAFLD and associated metabolic disorders when monocolonized in GF mice fed on HFD.

10.1128/mBio.03263-19.8 FIG S8 Different LPS-producing bacteria have different capacities to increase endotoxin load and to provoke systemic and local inflammation in the GF mice under HFD feeding. (A) Endotoxin activity of different LPS-producing bacteria, Klebsiella pneumoniae A7 (*Kleb*), Escherichia coli PY102 (*E.coli*), and the Bacteroides thetaiotaomicron type strain (*B.theta*), based on *Limulus* amebocyte lysate test. E. coli 055:B5 LPS (Sigma) was used as a positive control. (B) ELISA of serum LBP. (C) Population levels of LPS-producing bacteria strains in gnotobiotic mice. (D) ELISA of serum SAA-3. (E and F) RT-qPCR analysis of expression of *Tlr4*, *Cd14*, and *Lbp* in the liver (E) and epididymal fat pad (F). (G to I) RT-qPCR analysis of expression of *Tnfα*, *Il1β*, *Il6*, *Mcp1*, *Ikkε*, and *Reg3γ* in the liver (G), epididymal fat pad (H), or ileum (I). All mRNA quantification data were normalized against the housekeeping gene. Gene expression levels were expressed as values relative to those of the control group (HFD+LB). *Lbp*, LPS-binding protein gene; *Tlr4*, toll-like receptor 4 gene; *Tnfα*, tumor necrosis factor-α gene; *Il1β*, interleukin-1β gene; *Il6*, interleukin-6 gene; *Mcp1*, monocyte chemoattractant protein-1 gene; *Ikkε*, I kappa B kinase epsilon gene; *Reg3γ*, c-type lectin regenerating islet-derived protein 3-γ gene. Data are shown as means ± SEM (*n* = 7 to 16). *, *P < *0.05; **, *P < *0.01; ***, *P < *0.001. Download FIG S8, TIF file, 1.2 MB. Copyright © 2020 Fei et al. 2020 Fei et al. This content is distributed under the terms of the Creative Commons Attribution 4.0 International license.

10.1128/mBio.03263-19.9 FIG S9 Different LPS-producing bacteria had different capacities to induce obesity in GF mice under HFD feeding. (A) Growth curves. (B) Cecum content weight. (C) Cecum content weight (percentage of the body weight). (D to G) Serum lipid concentration. (H) Serum leptin concentration adjusted for body weight. (I to K) RT-qPCR analysis of the expression of *Leptin* in the epididymal fat pad (I), AccI, *Fas*, *Pparγ*, and *Scd1* in the liver (J), and *Angptl4* (*Fiaf*) in the ileum (K). (L) Total food intake under HFD feeding. (M) RT-quantitative PCR analysis of expression of *Glp1*, *Pyy*, and *Ghrelin* in the ileum. All mRNA quantification data were normalized against the housekeeping gene. Gene expression levels were expressed as values relative to those of the control group (HFD+LB). AccI, acetyl-CoA carboxylase-1 gene; *Fas*, fatty acid synthase gene; *Pparγ*, peroxisome proliferator-activated receptor-gamma gene; *Scd1*, stearoyl-CoA desaturase 1 gene; *Angptl4* (*Fiaf*), fasting-induced adipose factor gene. Data are shown as means ± SEM (*n* = 7 to 16). *, *P < *0.05; ***, *P < *0.001. Download FIG S9, TIF file, 1.3 MB. Copyright © 2020 Fei et al. 2020 Fei et al. This content is distributed under the terms of the Creative Commons Attribution 4.0 International license.

We next investigated whether a Gram-negative bacterium with nonproinflammatory LPS could also induce NAFLD in GF mice. Previous studies showed that LPS endotoxin activity of *Bacteroides* spp. was 1,000-fold lower than that of species in the *Enterobacteriaceae* family and failed to induce immune activation (34, 35). In light of this evidence, we introduced the commensal bacterium Bacteroides thetaiotaomicron type strain (ATCC 29148) to HFD-fed GF C57BL/6J mice (HFD+*B.theta*). The LAL test showed that LPS endotoxin activity from B. thetaiotaomicron was nearly 500-fold lower than that from K. pneumoniae A7, E. coli PY102, and E. cloacae B29 (Fig. S8) (36). HFD mice colonized by B. thetaiotaomicron exhibited slightly degenerated hepatocytes and significantly increased NAFLD activity scores compared with those of HFD GF mice but markedly lower than those of mice monocolonized with either strain K. pneumoniae A7 or E. coli PY102 (Fig. 4A and B). Furthermore, mice in group HFD+*B.theta* showed no obesity and an insulin-resistant phenotype (Fig. 4D to H and Fig. S9) (37). HFD+*B.theta* mice showed no significant difference in serum endotoxin load or local and systemic inflammatory levels from levels of the GF control, even though B. thetaiotaomicron reached a significantly higher population level in the gut of gnotobiotic mice than either of the other two proinflammatory bacterial strains (Fig. S8). These results further indicate that high endotoxin activity is essential for the NAFLD-inducing function of Gram-negative pathobionts.

The increasing incidence of NAFLD is widely thought to result from nutrient excess due to increased food consumption. We next investigated whether changes in food intake contribute to the NAFLD-inducing effect of gut Gram-negative pathobionts in GF mice. Notably, neither food intake nor gene expression patterns associated with the appetite-regulating peptides (ghrelin, peptide YY, and glucagon-like peptide-1) exhibited differences between mice with and without NAFLD phenotypes on HFD in all the animal trials (Fig. S2L to N and S^9^L and M). Therefore, the NAFLD-inducing effect of these LPS-producing gut pathobionts is independent of intake of HFD.

## DISCUSSION

During the last 2 decades, major advances have been made in understanding NAFLD’s association with gut microbial dysbiosis, providing novel opportunities as well as challenges in both the pathogenesis and treatment options of NAFLD (2). However, the questions of whether specific microbial strains or their consortium can causally trigger NAFLD and become possible therapeutic targets of NAFLD have not been mechanistically addressed. This is of particular interest and importance, since the whole gut microbiota cannot be the targets controlling liver disease (14). Using a monoassociated gnotobiotic model, our previous data support a crucial role for one endotoxin-producing strain, Enterobacter cloacae B29, in driving aggressive obesity (23). In the present study, by expanding the previously used approach, we identified that nonvirulent endotoxin-producing strains of pathobiont species overgrowing in the human gut can work as causative agents with LPS-TLR4 cross talk as the most upstream and essential molecular event for NAFLD and related metabolic disorders.

Until the present study, there has been limited opportunity to explore the putative link between the overgrowing of specific bugs, hepatic fat accumulation, and the development of NAFLD (38). Gram-negative bacteria have been shown to be associated with the progression of NAFLD in cohort studies (10, 11). In the present study, only Gram-negative bacteria belonging to the family *Enterobacteriaceae* with proinflammatory endotoxin (39), like Enterobacter cloacae B29, Klebsiella pneumoniae A7, and Escherichia coli PY102, could induce NAFLD and related metabolic diseases. Conversely, the strains belonging to the family *Bacteroidaceae* that do not have proinflammatory endotoxin, like the Bacteroides thetaiotaomicron type strain, exhibited only slightly degenerated hepatocytes compared to those of HFD GF mice. Findings on the effect of *Bacteroides* on the development of metabolic diseases were conflicting in the literature (37). Coinoculation of GF mice with Bacteroides thetaiotaomicron and Methanobrevibacter smithii has been shown to increase the epididymal fat pad but not the total body weight (37). However, Bacteroides thetaiotaomicron was also shown to be markedly decreased in obese individuals and alleviated diet-induced body weight gain and adiposity in mice (40). In this study, we have identified specific noninfectious, endotoxin-producing bacteria as causative agents for the development of NAFLD in humans by following the logic of Koch’s postulates (41, 42). First, these bacteria were selected as candidate pathogenic gut bacteria for inducing human NAFLD and related metabolic diseases, because they overgrew as dominant populations before dietary intervention and reduced to almost nondetectable levels soon after the intervention, with the fatty liver phenotype being significantly alleviated (41, 42). For example, in our previous studies, *Enterobacter*, a genus of opportunistic, endotoxin-producing pathogens (39), made up 35% of the total gut bacteria in a morbidly obese volunteer, and this level was reduced to nondetectable after 23 weeks on a diet composed of whole grains, traditional Chinese medicinal foods, and prebiotics (WTP diet), which led to a 51.4-kg weight loss along with the significantly alleviated fatty liver phenotype (23). Similarly, E. coli was detected as 40% of the gut microbiota in an obese 3-year-old girl. She lost 16 kg after 20 weeks with alleviated fatty liver phenotype on the WTP diet, and the E. coli population was reduced to nondetectable soon after starting the diet. These LPS-producing strains were predominant members of the gut microbiota when their hosts were obese and had fatty liver phenotypes (23). Second, pure cultures of these candidate bacteria were isolated from the human donor samples; in our study, Enterobacter cloacae B29 was isolated from the baseline fecal sample of the adult volunteer as the most abundant pathobiont (23). Klebsiella pneumoniae A7 was isolated from the same volunteer’s gut as the second most abundant pathobiont (23). Escherichia coli PY102 was isolated from the baseline sample of a 3-year-old girl. Third, monocolonization of pure culture of these candidate bacteria in GF mice on HFD reproduced NAFLD and other related phenotypes in metabolic syndromes. In the present study, all three Gram-negative bacteria, Enterobacter cloacae B29, Klebsiella pneumoniae A7, and Escherichia coli PY102, could induce NAFLD and related metabolic diseases in GF mice under HFD feeding. Finally, genetic mutations from both the candidate bacteria and host showed that LPS-TLR4 cross talk is required for the initiation and progression of all NAFLD-related phenotypes. Our study provides definitive evidence that specific endotoxin-producing gut bacteria work as causative agents (pathogens) for NAFLD development in humans.

Understanding the mechanisms by which the specific gut microbes influence the progression of NAFLD may allow us to establish gut microbiota-targeted precision medicine in the treatment of NAFLD. At the molecular level, LPS is recognized by the specific pattern recognition receptor TLR4 (43, 44) and its coreceptors, LBP and CD14 (21, 22), which triggers a downstream inflammatory cascade (21, 45). TLR4-deficient mice display decreased liver injury, inflammation, and lipid accumulation compared with wild-type mice in NAFLD models induced by high fructose or NCD diet (28, 46), which confirms the role of TLR4 in inducing inflammation in NAFLD. In accordance with previous reports, our study shows that the blocking of the LPS-TLR4 interaction between gut pathobionts and host cells can prevent the initiation and development of NAFLD and all other obesity-related phenotypes otherwise inducible on HFD. This indicates that the LPS-TLR4 pathway works as the most upstream molecular event in the specific endotoxin-producing gut bacteria-NAFLD cross talk, consistent with previous research (17, 18). Chronic systemic inflammation driven by these abnormally prevalent pathobiont populations then drives the development of NAFLD and obesity in human hosts (19). Although LPS endotoxin production is essential for these pathobionts to induce NAFLD, as shown in our study, it may not be sufficient. In the complex gut ecosystem, these pathobionts still need to outcompete other bacteria and evade host immune reactions so they can establish a sufficiently higher population level than usual for a long enough time for their proinflammatory properties to come into effect (14, 47).

Our study has some limitations. Except for LPS, other gut microbe-derived components or metabolites, such as peptidoglycans (48), trimethylamine (49), secondary bile acids (50), short-chain fatty acids (51), branched-chain amino acids (52), and ethanol (53), have also been proven to contribute to the regulation of hepatic fat accumulation and the pathogenesis of NAFLD. There have also been many reports of increased intestinal permeability with NAFLD (53–55), which might be an important contributor to disease progression, even though these results were not confirmed in all studies (55). In addition to the LPS-TLR4 pathway, other molecular pathways are involved in the development of NAFLD, and their modulation by specific gut bacteria remains to be established. Adipose tissue also has significant effects on the development of NAFLD (56). For example, the excessive fat deposition in the liver could be initiated by increased fatty acid delivery from adipose tissue (57); hypertrophic and hypoxic adipocytes develop an inflammatory phenotype, secreting inflammatory cytokines into circulation, which induces hepatocyte death and modulates hepatic immune function (58). In our current study, HFD+B29/*Kleb*/*E.coli* gnotobiotic mice had the greatest increase in triglyceride content, suggesting that these mice had the greatest increase in hepatic steatosis; however, the plasma triglyceride concentrations were not modified. Our current study provides *in vivo* evidence to show the causal relationship between the special gut microbes and the development of NAFLD and other related metabolic diseases. However, the molecular mechanisms on the host side underlying the LPS-producing bacteria inducing these metabolic derangements still need further deep investigation. Apolipoprotein B (ApoB) is important in the export of triglycerides from the liver and prevents fatty liver conditions (59). More studies are required to define further the gut microbes that may have a particularly important role in determining the lipid-transporting capability of the ApoB molecule or mitochondrial β oxidation in the liver. Distinct microbiome signatures may be associated with different stages of liver diseases, and specific pathobionts may serve as noninvasive microbiota-based predictive or diagnostic biomarkers after their causative role in disease progression has been demonstrated.

In summary, it is imperative to identify NAFLD-related microbes at the strain level. Drawing any clear conclusions on the role of specific bacteria or their consortium in the onset and development of NAFLD has to happen before novel therapy can be developed. Actually, identification of specific gut bacteria mechanistically causing or driving NAFLD is currently in its infancy. Importantly, our data point to a direct pathogenetic role of endotoxin-producing gut bacteria and their molecular cross talk with the host. To the best of our knowledge, this is the first evidence *in vivo* that mechanically shows the association between specific gut taxa and NAFLD. While their implications remain to be refined, the present findings open new avenues for managing NAFLD and related metabolic diseases worldwide.

## MATERIALS AND METHODS

### Animal experimental design.

Germfree (GF) male C57BL/6J mice (provided by Anaxem, the GF animal facility of the Micalis Institute, INRA, Jouy-en-Josas, France), C3H/HeN wild-type mice (provided by Anaxem), and C3H/HeN TLR4^−/−^ mice (kind gift from the Animal Resources Center, the University of Chicago) were bred under the GF rodent breeding conditions at Anaxem. All mice were maintained on a 12 h-12 h lighted-dark cycle and first supplied with a 45-kGy irradiated sterile pelleted normal chow diet (NCD; energy content of 13.5% fat, 25.2% protein, and 61.3% carbohydrate; standard maintenance diet R03-40; SAFE, France) or high-fat diet (HFD; energy content of 60% fat, 20% protein, and 20% carbohydrate; D12492; Research Diets, Inc., New Brunswick, NJ) and autoclaved tap water *ad libitum*. GF C57BL/6J mice (6 to 8 weeks old) were divided into 4 groups randomly. E. cloacae B29 and B29Δ*waaG* strains, Bacteroides thetaiotaomicron, Klebsiella pneumoniae, or Escherichia coli monocolonized mice were obtained by a single intragastric gavage with 10^9^ to 10^10^ cells of B29 and B29Δ*waaG* strains, the B. thetaiotaomicron type strain (ATCC 29148), K. pneumoniae A7, or E. coli PY102 per mouse in 0.1 sterile medium fed separately on NCD or HFD. The GF control group was treated with 0.1 ml sterile LB medium per mouse. Each mouse group was raised in a separate isolator and fed on HFD for 15 weeks.

For all animal experiments, body weight and food intake were monitored weekly with an electronic weight indicator (Dini Argeo, Italy). Mice were sacrificed by cervical dislocation, and tissues were collected. Sections of epididymal adipose tissue and the main lobe of liver were fixed in 4% paraformaldehyde–phosphate-buffered saline (PBS) for 48 h and then washed and stored in 70% ethanol. All other sections of the tissues were stored in either liquid nitrogen or RNAlater (Ambion) immediately after exsanguination and stored at –80°C until processing. Details are presented in the supplemental material.

### Bacterial strains.

Enterobacter cloacae B29, K. pneumoniae A7, and E. coli PY102 were grown in LB broth or on LB agar plates at 37°C. The B. thetaiotaomicron type strain (ATCC 29148) was cultured in *Bacteroides* bile esculin (BBE) broth at 37°C under anaerobic conditions.

To determine the colonization levels of the bacteria in the gut, gnotobiotic mice were checked at 1 week, 4 weeks, 8 weeks, 12 weeks, and 15 weeks after inoculation by plating appropriate serial dilutions of feces on the LB or BBE agar plates and counting the colonies after incubation. DNA extraction from feces and 16S rRNA gene PCR sequencing were performed to confirm the presence of one bacterium only in monocolonized animals (data not shown).

### Construction of the *waaG* mutant of Enterobacter cloacae B29.

A *waaG* deletion mutant of Enterobacter cloacae B29 was constructed using a positive-selection suicide vector, pKNG101 (60), which contains a *pir*-dependent R6K origin of replication (oriR6K), the *strAB* gene encoding the streptomycin phosphotransferase (Sm^r^), an origin of transfer (mobRK2), the *sacB* gene mediating sucrose sensitivity (Suc^s^), and multiple cloning sites. Briefly, a 619-bp DNA fragment upstream and 651-bp DNA fragment downstream of the target gene *waaG* were PCR amplified using the oligonucleotides pairs WGPA/WGPB (5′-CTAGGGCCCGATTGACCGCATT-3′/5′-GTTAGGGAAAGGATCATACGTTCGTGGCTCTGGAC-3′) and WGPC/WGPD (5′-CAATCCCTTTCCTCAATCCCTTTCCTCGAGACCTG-3′/5′-CTAGGGCCCGGAGAACATCCCGTGT-3′), respectively. The oligonucleotides WGPB and WGPC were designed for amplifying the two fragments with overlapping 3′ and 5′ ends to facilitate splicing by overlapping extension (SOE). Agarose gel-purified fragments upstream and downstream of *waaG* were ligated by performing an overlapping PCR. The resulting PCR product without the 574-bp *waaG* fragment was then cloned into the pKNG101 suicide vector. The constructs were transformed into E. coli SM10 *λpir* as a donor strain via electroporation, followed by mobilization into wild-type strain B29 by conjugal mating. Integration of the suicide vector results in an Sm^r^ and Suc^s^ phenotype. B29/*pKNG101* recombination cells with a single chromosomal integration event were selected on agar plates containing streptomycin (50 μg ml^−1^) and rifampin (100 μg ml^−1^) to select against the E. coli SM10 *λpir/*pKNG101 donor because of the rifampin-resistant character of B29. Sm^r^ Suc^s^ cells were selected and plated onto LB plates containing 10% sucrose to select cells with a second homologous recombination. Finally, Sm^s^ Suc^r^ cells were confirmed to contain the gene deletion by PCR and sequencing. The resulting mutant was designated the B29Δ*waaG* strain and was indistinguishable from the wild-type strain, except for the loss of a 574-bp *waaG* DNA fragment.

### LPS extraction and endotoxin activity detection.

LPS extraction from Enterobacter cloacae B29, the B29Δ*waaG* mutant, the B. thetaiotaomicron type strain (ATCC 29148), K. pneumoniae A7, or Escherichia coli PY102 was performed using the LPS extraction kit (iNtRON Biotechnology Co., Seoul, South Korea) according to the manufacturer’s instructions. Endotoxin activity analyses were performed using the chromogenic substrate *Limulus* amebocyte lysate (LAL) assay (Associates of Cape Cod Incorporated, USA). The LPS derived from E. coli 055:B5 (L2880; phenol extract; Sigma) was used as a positive control in the assay.

### Oral glucose tolerance test and assessment of insulin secretion.

Mice were fasted for 5 h, and baseline blood glucose levels were measured with an ACCU-CHEK Performa (Roche) glucometer using blood collected from the tail vein. Glucose (2 g/kg body weight) was given orally by gavage, and blood glucose levels then were measured 15, 30, 60, 90, and 120 min after gavage. Blood was sampled before and 30 min after the glucose load to assess plasma insulin levels via enzyme-linked immunosorbent assay (ELISA).

### Serum biochemical analysis.

Serum insulin, leptin, adiponectin, and SAA-3 levels were determined by ELISA kits from Millipore Corporation (Billerica, MA). The serum LBP level was measured by a mouse lipopolysaccharide binding protein ELISA kit (HyCult Biotechnology, Uden, The Netherlands). All assays were performed according to the manufacturers’ instructions.

### Liver triglyceride extraction and measurement.

Portions of frozen liver from receiver mice were homogenized in chloroform-methanol (2:1) to extract total lipids according to the methodology of Folch et al. (61). The organic extract was dried and reconstituted in isopropanol. The triglyceride content was measured with a triglyceride’s determination kit (Sigma-Aldrich, Saint-Louis, MO, USA) according to the manufacturer’s instructions.

### Epididymal adipose tissue histology.

Adipose tissue morphometric analysis was performed on the sections stained by hematoxylin-eosin-saffron (HES stain). Slides were digitalized using a Pannoramic digital slide scanner (3DHistech Ltd., Budapest, Hungary), and adipocyte morphometry was analyzed, quantified, and photographed using Pannoramic Viewer software by 3DHistech. The number of adipocytes per microscopic field (at least 5 fields per tissue) was determined at a magnification of ×200, and the average surface area of the adipocytes (in square micrometers) was calculated.

### TaqMan low-density array RT-qPCR assay and data analysis.

Quantitative real-time PCR (RT-qPCR) utilized a custom-made TaqMan low-density array RT-qPCR assay (TLDA) cards from Life Technologies (Thermo Fisher Scientific), by following the manufacturer’s instructions, to separately examine the expression levels of 21 selected mouse genes in liver, ileum, or epididymal adipose tissue, which may be involved in metabolic syndrome development, and 3 housekeeping genes (glyceraldehyde-3-phosphate dehydrogenase [GAPDH], 18S, and ACTB) (see Table S1 in the supplemental material). In this study, the TLDA cards were configured into eight identical 24-gene sets (duplicate per sample). Briefly, each cDNA (30 μl) sample was mixed with 25 μl of H_2_O and 55 μl of 2× TaqMan universal PCR master mix (Applied Biosystems). Each sample (100 μl PCR mixture) then was loaded into each pool of the TLDA card. Thermal cycling was performed using an Applied Biosystems 7900HT sequence detection system for 2 min at 50°C and 10 min at 95°C, followed by 40 cycles for 15 s at 95°C and 1 min at 60°C. TLDA data were collected with the manufacturer’s SDS software. RQ Manager Analysis software (Applied Biosystems) was used to process the array data and determine the threshold cycle (*C_T_*) values. Thresholds, set at 0.2, were checked individually and corrected as necessary. GAPDH and 18S genes were chosen for this study, as they were identified as the least variable of all housekeeping genes included in the TLDA assays. Consequently, data were normalized against the housekeeping gene using the values of each of the 8 pools. Relative quantification of gene expression was determined using the comparative *C_T_* method, which means the fold change in gene expression in the treated group relative to the control was calculated by the equation 2^−ΔΔ^*^CT^* = 2^–[(^*^CT^* ^target gene –^ *^CT^* ^GAPDH)treated – (^*^CT^* ^target gene –^ *^CT^* ^GAPDH)untreated]^ (62).

10.1128/mBio.03263-19.10 TABLE S1 The target genes and assay identifiers of the primer probes used in the TaqMan gene expression assays for liver, ileum, or epididymal adipose tissue. Download Table S1, DOCX file, 0.03 MB. Copyright © 2020 Fei et al. 2020 Fei et al. This content is distributed under the terms of the Creative Commons Attribution 4.0 International license.

### Statistical analysis.

Statistical analysis was performed using SPSS statistical software package 17.0 (SPSS, Chicago, IL, USA). Normal distribution of the data was determined using the Shapiro-Wilk test. A one-way analysis of variance (ANOVA) followed by a *post hoc* (Turkey’s multiple-comparison test) was used for data between multiple groups that were normally distributed, and the Kruskal-Wallis H-test was used for data that were not normally distributed. Student's *t* test (normally) or Mann-Whitney U test (not normally) was used for comparing differences between two groups. Results are expressed as means ± standard errors of the means (SEM), with statistical significance set at 0.05, 0.01, or 0.001.

The clinical study was approved by the Ethics Committee of Chinese Clinical Trial Registry (registration number ChiECRCT-000011).
