# Supplementary figures and images for: Endotoxin Producers Overgrowing in Human Gut Microbiota as the Causative Agents for Nonalcoholic Fatty Liver Disease
Source: mBio. 2020 Feb 4;11(1):e03263-19. doi: 10.1128/mBio.03263-19 (PMC7002352; doi:10.1128/mBio.03263-19)

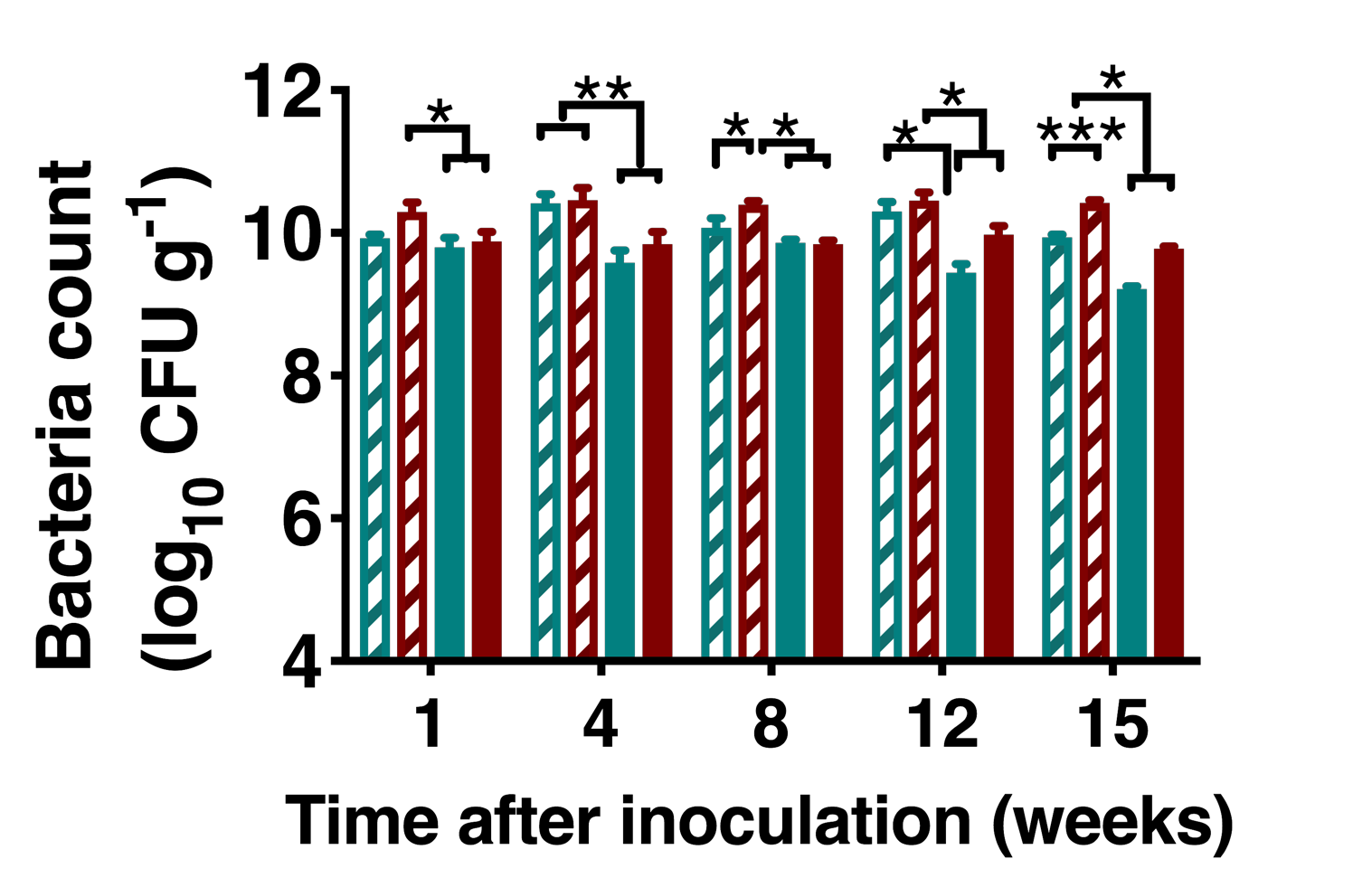

Supplement: FIG S1 [file mBio.03263-19-sf001.tif]

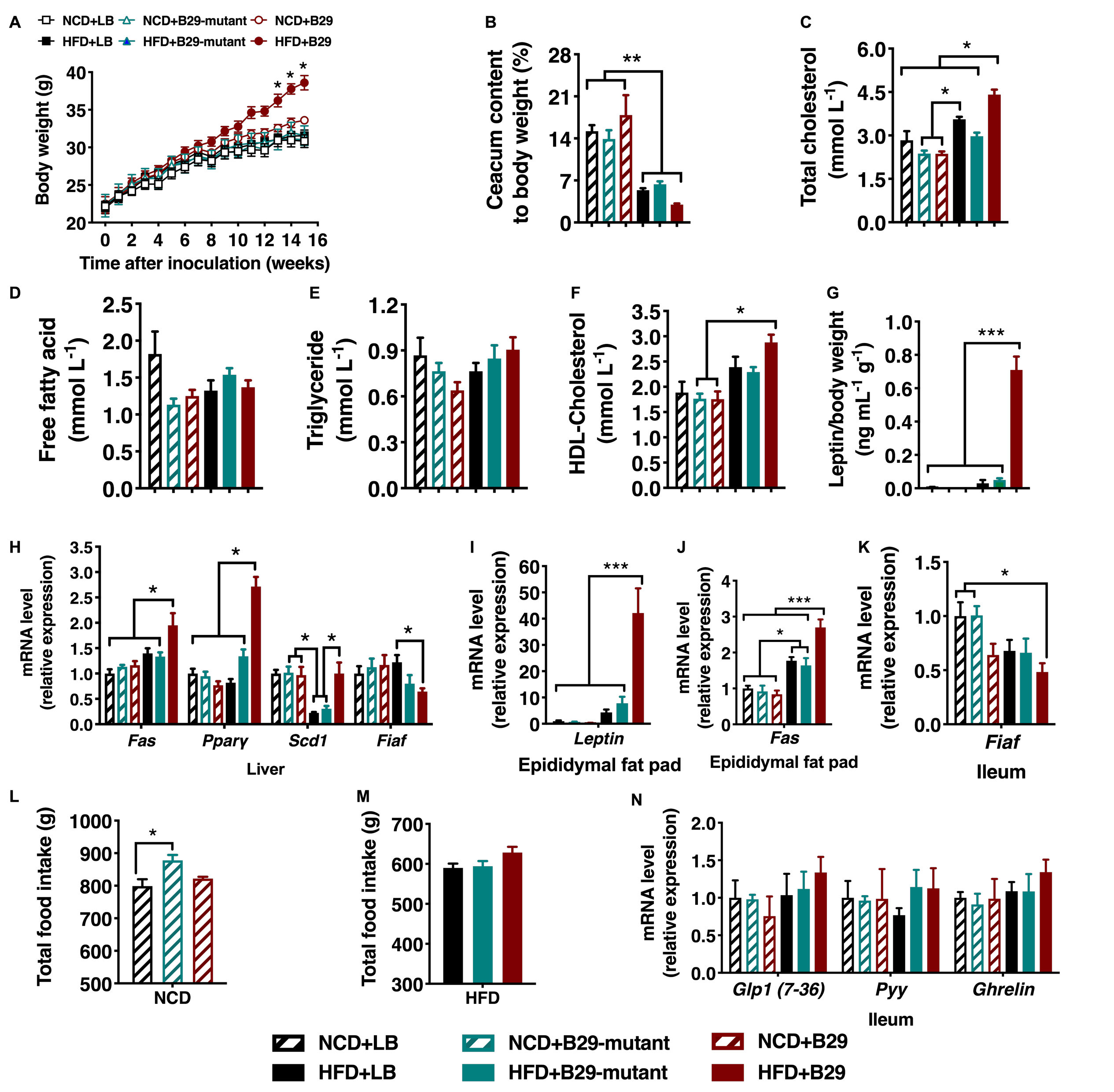

Supplement: FIG S2 [file mBio.03263-19-sf002.tif]

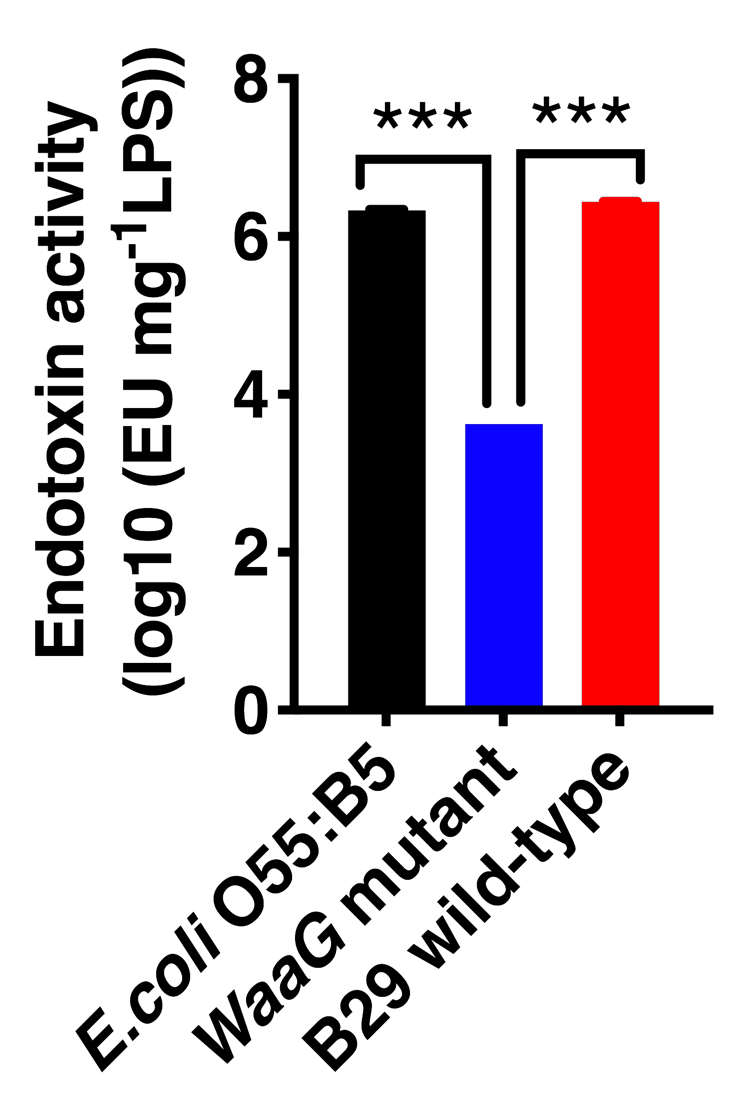

Supplement: FIG S3 [file mBio.03263-19-sf003.tif]

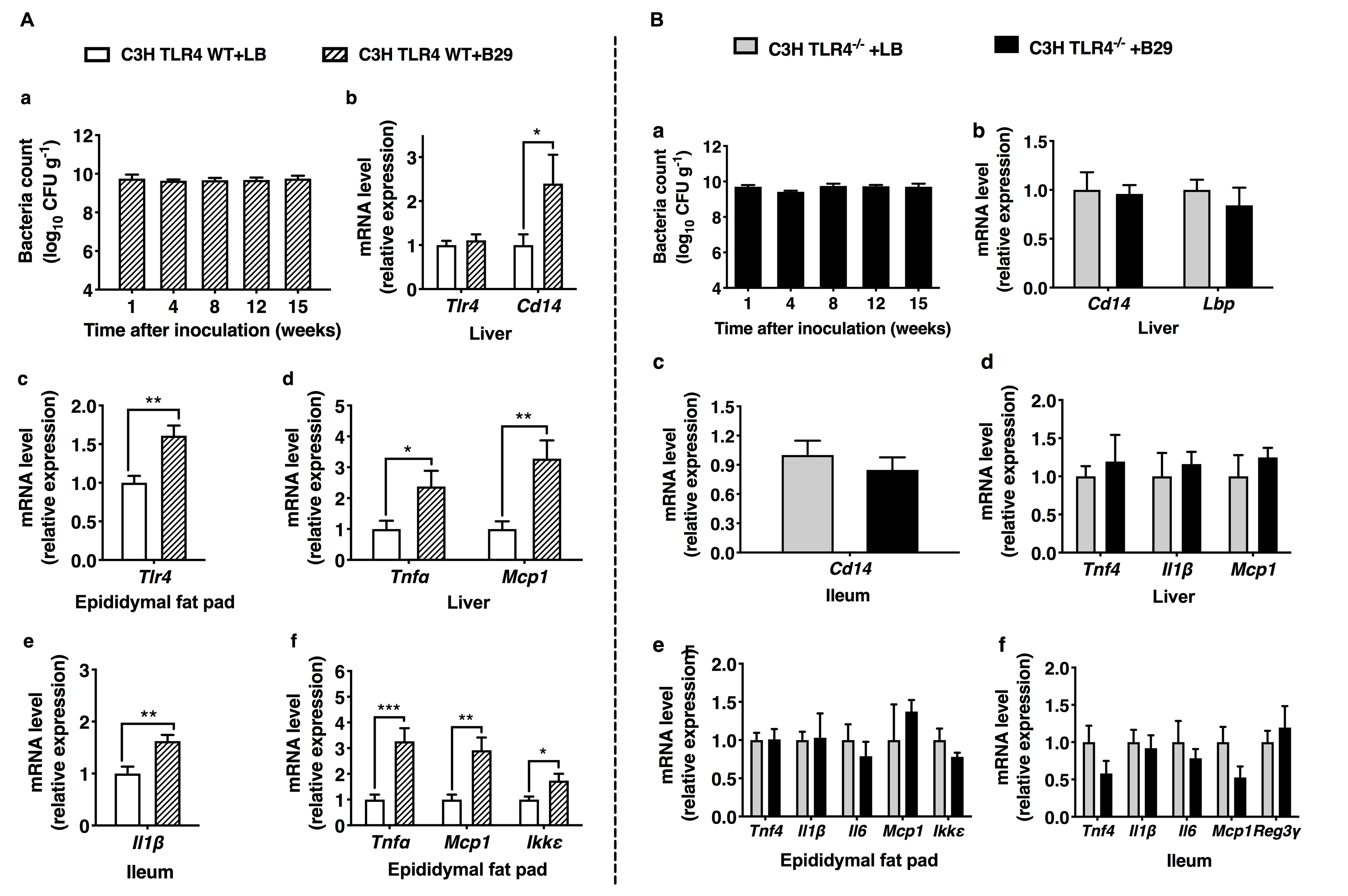

Supplement: FIG S4 [file mBio.03263-19-sf004.tif]

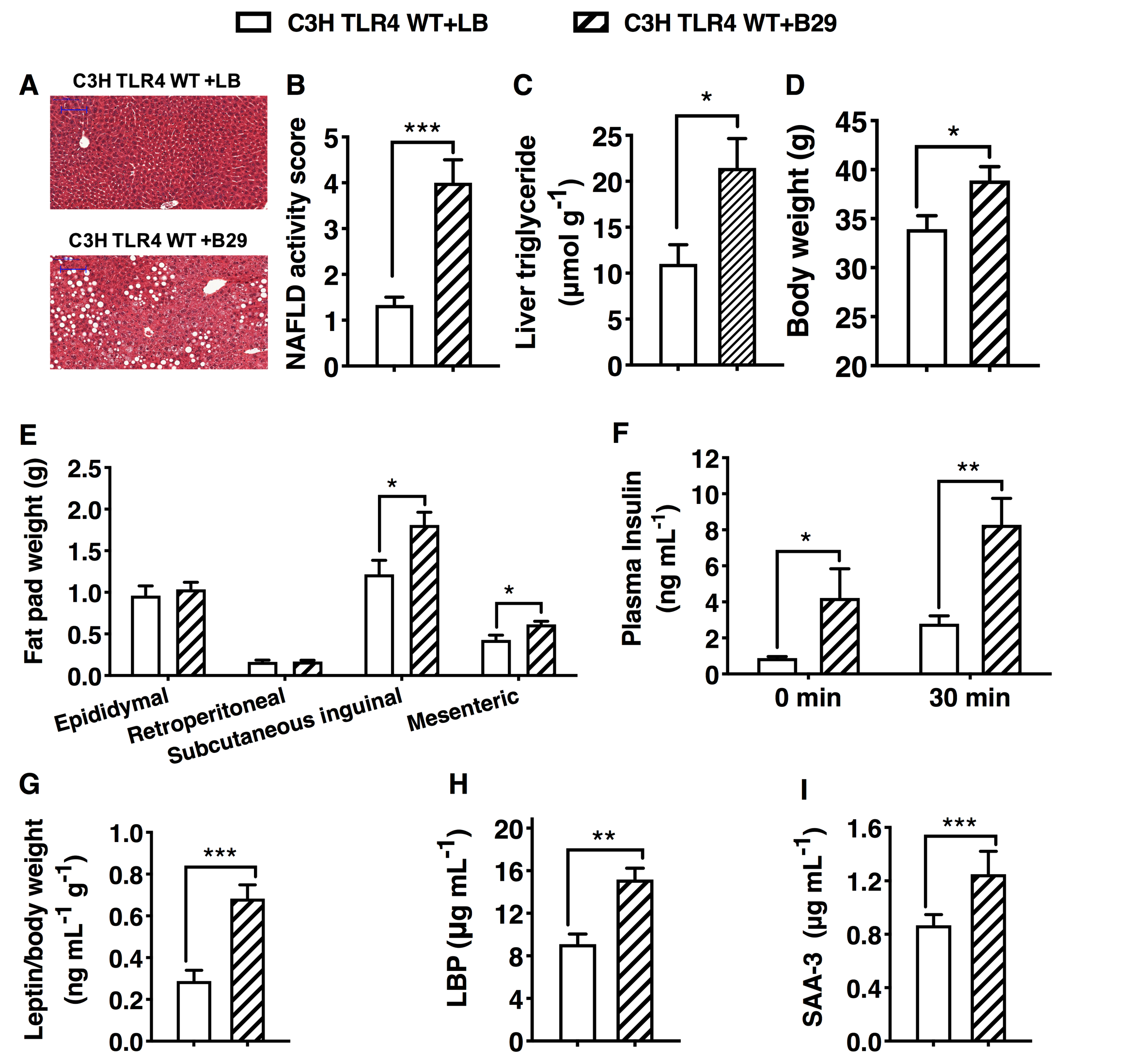

Supplement: FIG S5 [file mBio.03263-19-sf005.tif]

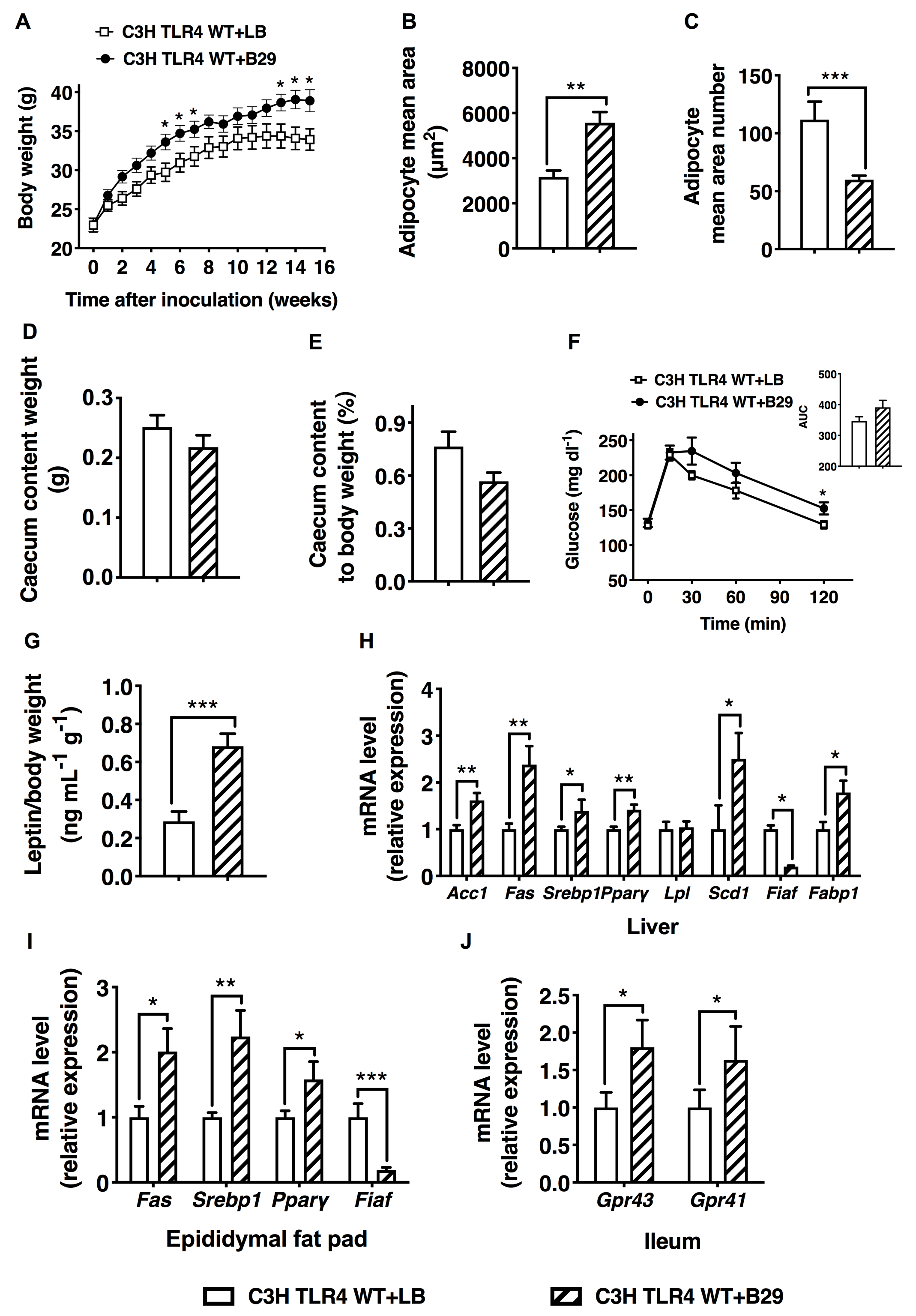

Supplement: FIG S6 [file mBio.03263-19-sf006.tif]

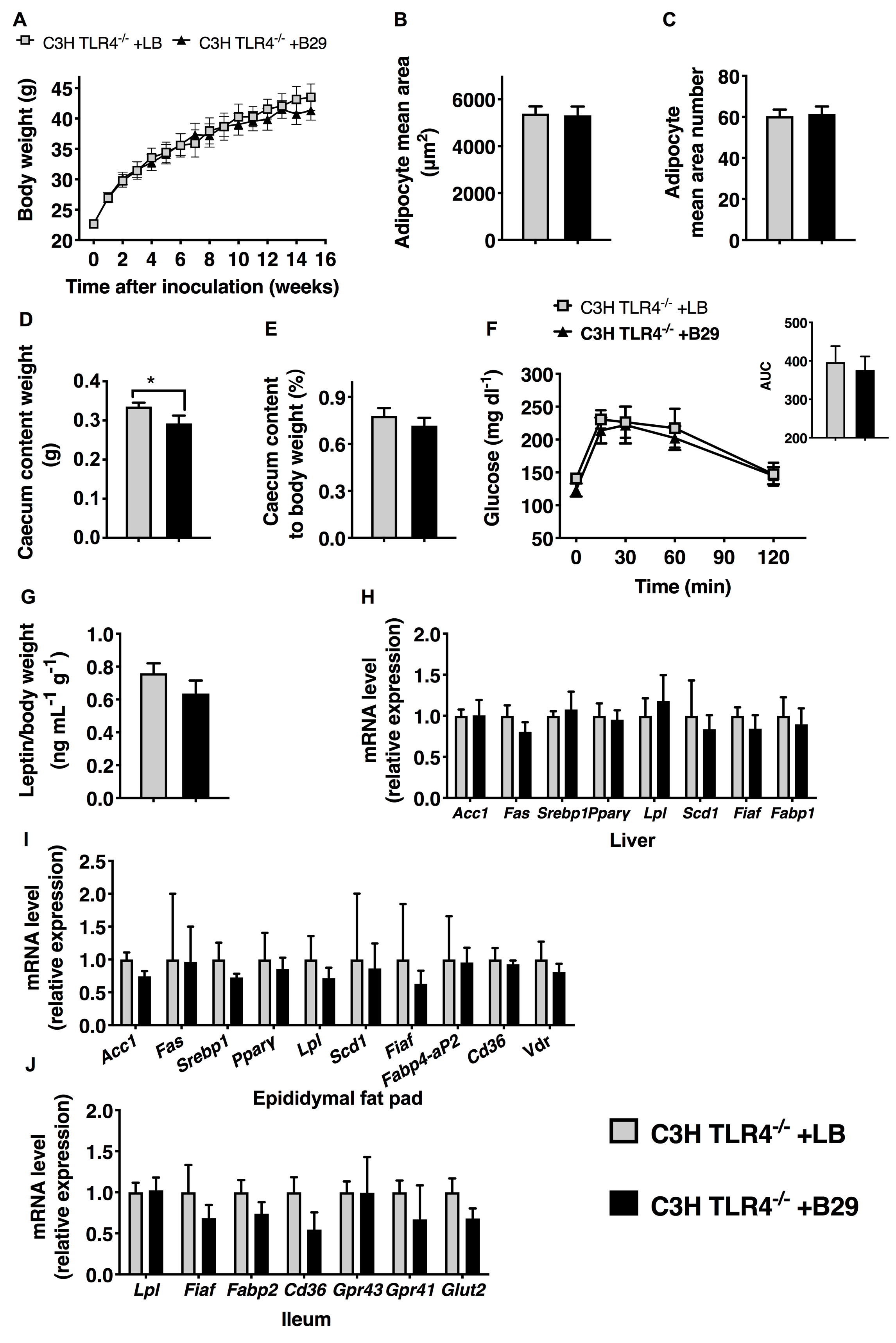

Supplement: FIG S7 [file mBio.03263-19-sf007.tif]

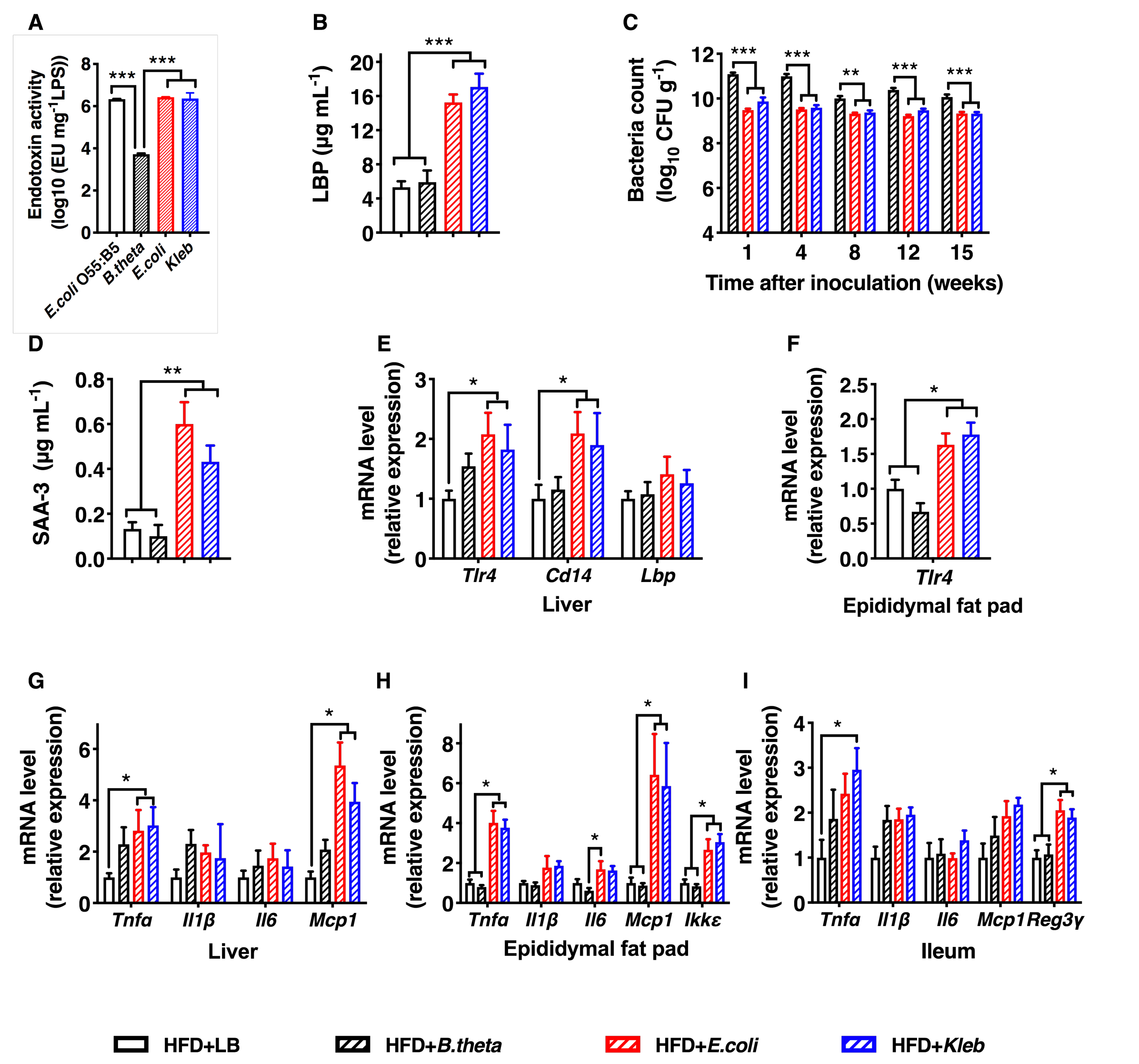

Supplement: FIG S8 [file mBio.03263-19-sf008.tif]

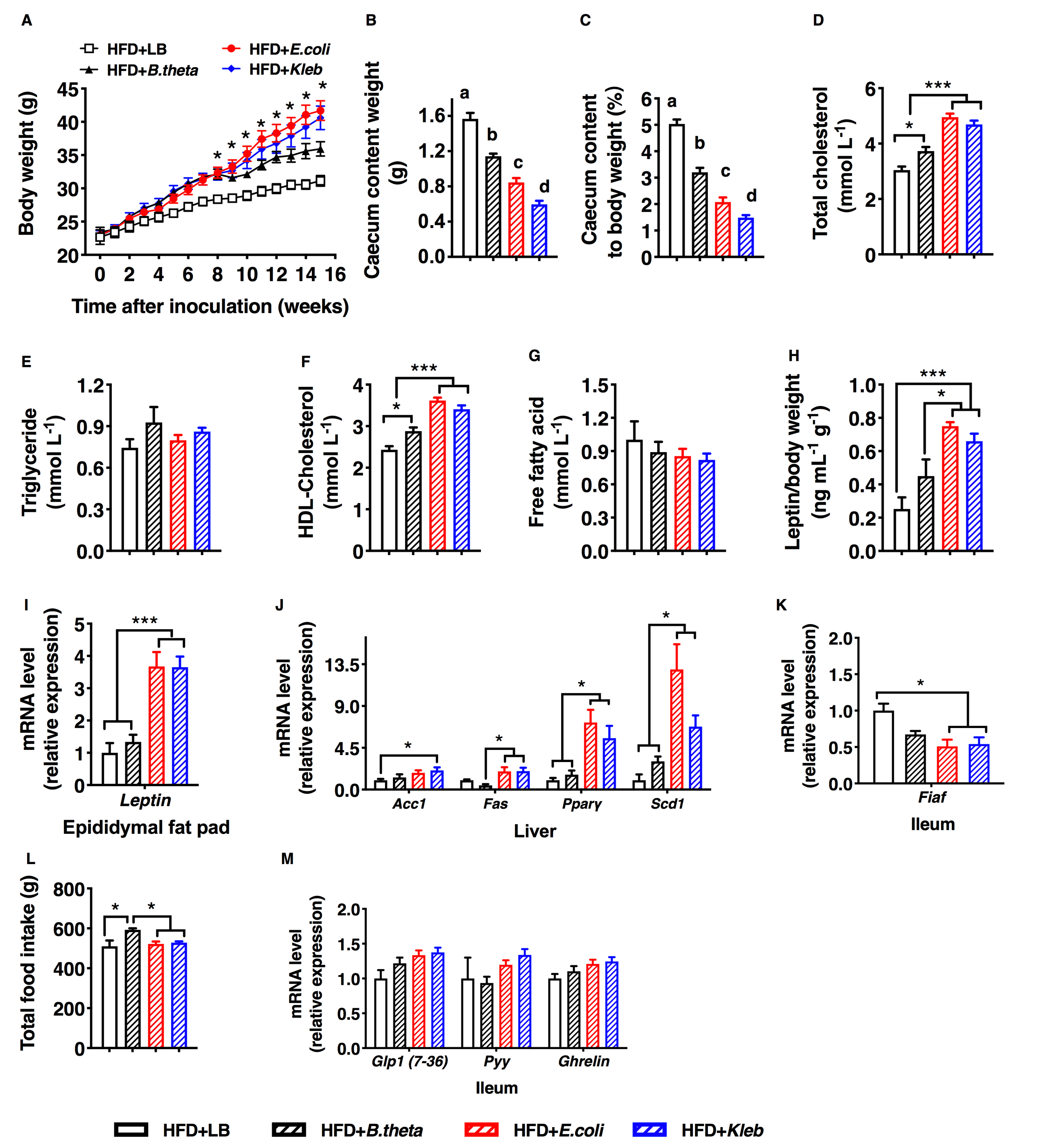

Supplement: FIG S9 [file mBio.03263-19-sf009.tif]
