# Supplementary material for: Endotoxin Producers Overgrowing in Human Gut Microbiota as the Causative Agents for Nonalcoholic Fatty Liver Disease
Source: mBio. 2020 Feb 4;11(1):e03263-19. doi: 10.1128/mBio.03263-19 (PMC7002352; doi:10.1128/mBio.03263-19)
Supplement: TABLE S1 [file mBio.03263-19-st001.docx]

**Table S1.** **The target genes and assay IDs of the primer probes used in the TaqMan gene expression assays for liver, ileum or epididymal adipose tissue.**

| **Gene symbol** | **Assay ID** | **Liver** | **Epididymal fat pat** | **Ileum** |
| --- | --- | --- | --- | --- |
| *Tlr4* | Mm00445273_m1 | √ | √ | √ |
| *Lbp* | Mm00493139_m1 | √ |  |  |
| *Cd14* | Mm00438094_g1 | √ |  | √ |
| *Tnfα* | Mm00443258_m1 | √ | √ | √ |
| *Il1β (Il1b)* | Mm00434228_m1 | √ | √ | √ |
| *Il6* | Mm00446190_m1 | √ | √ | √ |
| *Mcp1 (Ccl2)* | Mm00441242_m1 | √ | √ | √ |
| *Ikkε (Ikbke)* | Mm00444862_m1 |  | √ |  |
| *Reg3γ* | Mm01181783_g1 |  |  | √ |
| *Zo1 (Tjp1)* | Mm00493699_m1 |  |  | √ |
| *Occludin (Ocln)* | Mm00500912_m1 |  |  | √ |
| *Glp-2r* | Mm01329475_m1 |  |  | √ |
| *Acc1 (Acaca)* | Mm01304257_m1 | √ | √ |  |
| *Fas (Fasn)* | Mm00662319_m1 | √ | √ |  |
| *Srebp1* | Mm00550338_m1 | √ | √ |  |
| *Pparγ (Pparg)* | Mm01184322_m1 | √ | √ |  |
| *Lpl* | Mm01345523_m1 | √ | √ | √ |
| *Scd1* | Mm00772290_m1 | √ | √ |  |
| *Angptl4 (Fiaf)* | Mm00480431_m1 | √ | √ | √ |
| *Fabp4-aP2* | Mm00445878_m1 |  | √ |  |
| *L-Fabp (Fabp1)* | Mm00444340_m1 | √ |  |  |
| *I-Fabp (Fabp2)* | Mm00433188_m1 |  |  | √ |
| *Cd36* | Mm01135198_m1 |  | √ | √ |
| *Gpr43 (Ffar2)* | Mm02620654_s1 |  |  | √ |
| *Gpr41 (Ffar3)* | Mm02621638_s1 |  |  | √ |
| *Glut2 (Slc2a2)* | Mm00446229_m1 |  |  | √ |
| *Vdr* | Mm00437297_m1 |  | √ |  |
| *Glp1 (7-36) (Gcg)* | Mm01269055_m1 |  |  | √ |
| *Pyy* | Mm00520716_g1 |  |  | √ |
| *Ghrelin (Ghrl)* | Mm00612524_m1 |  |  | √ |
| *Leptin (Lep)* | Mm00434759_m1 |  | √ |  |
| *Nfκb1* | Mm00476361_m1 | √ | √ | √ |
| *Myd88* | Mm00440338_m1 | √ |  |  |
| *Irs1* | Mm01278327_m1 | √ | √ |  |
| *Jnk1(Mapk8)* | Mm00489514_m1 | √ | √ |  |
| *Adiponectin (Adipoq)* | Mm00456425_m1 |  | √ |  |
| *Timp1* | Mm00441818_m1 | √ |  |  |
| *Tgf-β1* | Mm01178820_m1 | √ |  |  |
| *18s* | Hs99999901_s1 | √ | √ | √ |
| *Actb* | Mm00607939_s1 | √ | √ | √ |
| *Gapdh* | Mm99999915_g1 | √ | √ | √ |
